# Metabolic Hormone FGF21 Is Induced in Ground Squirrels during Hibernation but Its Overexpression Is Not Sufficient to Cause Torpor

**DOI:** 10.1371/journal.pone.0053574

**Published:** 2013-01-02

**Authors:** Bethany T. Nelson, Xunshan Ding, Jamie Boney-Montoya, Robert D. Gerard, Steven A. Kliewer, Matthew T. Andrews

**Affiliations:** 1 Department of Biology, University of Minnesota Duluth, Duluth, Minnesota, United States of America; 2 Department of Molecular Biology, University of Texas, Southwestern Medical Center, Dallas, Texas, United States of America; University of California, San Francisco, United States of America

## Abstract

Hibernation is a natural adaptation that allows certain mammals to survive physiological extremes that are lethal to humans. Near freezing body temperatures, heart rates of 3–10 beats per minute, absence of food consumption, and depressed metabolism are characteristic of hibernation torpor bouts that are periodically interrupted by brief interbout arousals (IBAs). The molecular basis of torpor induction is unknown, however starved mice overexpressing the metabolic hormone fibroblast growth factor 21 (FGF21) promote fat utilization, reduce body temperature, and readily enter torpor–all hallmarks of mammalian hibernation. In this study we cloned FGF21 from the naturally hibernating thirteen-lined ground squirrel (*Ictidomys tridecemlineatus*) and found that levels of FGF21 mRNA in liver and FGF21 protein in serum are elevated during hibernation torpor bouts and significantly elevated during IBAs compared to summer active animals. The effects of artificially elevating circulating FGF21 concentrations 50 to 100-fold via adenoviral-mediated overexpression were examined at three different times of the year. This is the first time that a transgenic approach has been used in a natural hibernator to examine mechanistic aspects of hibernation. Surgically implanted transmitters measured various metrics of the hibernation phenotype over a 7-day period including changes in motor activity, heart rate and core body temperature. In April fed-state animals, FGF21 overexpression decreased blood insulin and free fatty acid concentrations, effects similar to those seen in obese mice. However, elevated FGF21 concentrations did not cause torpor in these fed-state animals nor did they cause torpor or affect metabolic parameters in fasted-state animals in March/April, August or October. We conclude that FGF21 is strongly regulated during torpor and IBA but that its overexpression is not sufficient to cause torpor in naturally hibernating ground squirrels.

## Introduction

Hibernation is characterized by physiological changes that cause certain mammals to enter a state of suspended animation called torpor. In small hibernators such as ground squirrels torpor is characterized by heart rate rates of 3–10 beats per minute, body temperatures of 4–7°C, and oxygen consumption that is approximately 2% of the active state (reviewed in [1]). Studies aimed at identifying a substance that “triggers” this natural adaptation have failed to conclusively identify specific molecules that induce the hibernation phenotype [2]–[4]. Similarities of hibernation with starvation [5] and energy deprivation [6] in non-hibernating mammals have prompted us to search for regulatory proteins and other molecules that control analogous processes in natural hibernators. In this paper we examine a member of the fibroblast growth factor (FGF) family of proteins, fibroblast growth factor 21 (FGF21), as a potential regulator of hibernation in the thirteen-lined ground squirrel (*Ictidomys tridecemlinatus*).

As the name implies, many of the proteins in the FGF family stimulate cell growth and mitosis [7]. However a subfamily of the FGF proteins, including FGF19, 21 and 23, act as endocrine hormones to regulate bile acid homeostasis, glucose and lipid metabolism, and phosphate and vitamin D homeostasis, respectively [8]–[10]. Human FGF21 shows closest sequence similarity to FGF19, with approximately 35% amino acid identity [11]. FGF21 is expressed in the liver [11] and is involved in regulation of the balance between lipolysis and glycolysis, notably during fasting or a ketogenic diet [10], [12]. During fasting, activation of PPARα leads to an increase in liver FGF21 mRNA levels with concomitant elevation of FGF21 protein in the blood [12], [13]. FGF21 binds fibroblast growth factor receptors (FGFR) -1c, -2c, and -3c, with the strongest interactions with FGFR-1c in the presence of another protein, βKlotho [14]–[17]. βKlotho is a necessary component for FGF21 binding to the FGF receptors and is also required for downstream signaling [15]–[18].

In mice FGF21 has been shown to be involved in several physiological changes that resemble the transition from the active to torpid state in natural hibernators. Under starvation conditions transgenic mice overexpressing FGF21 readily enter torpor [13]. FGF21 lowers serum glucose [19], [20] and increases serum levels of the ketone D-β-hydroxybutyrate (BHB) [13]. This reciprocal change in metabolite concentrations is also seen in ground squirrels during hibernation [21]. Other similarities with hibernation include increases in circulating free fatty acids and glycerol, and decreases in serum triacylglycerols [13]. FGF21 is also capable of crossing the blood brain barrier [22] suggesting that it may have activities regulating neural control of the torpor response in fasting mice, and potentially regulating the extreme physiology that a hibernator experiences seasonally.

Various small molecules have been shown to induce short-term torpor in mice (reviewed in [23]), and recently adenosine A(1) receptors were shown to be involved with the induction of torpor in Arctic ground squirrels [24], but identification of a blood-born substance that induces torpor in hibernators has been elusive. In this study, we hypothesized that FGF21 is released into the blood where it circulates throughout the body initiating metabolic rate reduction, fat catabolism and torpor. To test this hypothesis we cloned the thirteen-lined ground squirrel FGF21 and characterized its expression at various points during the hibernation season. We subsequently employed adenovirus-mediated overexpression of ground squirrel FGF21 to examine the physiologic effects of elevating this hormone under different nutritional and seasonal conditions. This study is first time that a transgenic approach has been used in a natural hibernator to examine mechanistic aspects of the hibernation phenotype.

## Materials and Methods

### Animal Maintenance and Surgery

All animal use in this study was carried out in strict accordance with the approval of the University of Minnesota Institutional Animal Care and Use Committee (protocol #0707A12902). Thirteen-lined ground squirrels were wild-caught in central Minnesota and transferred to Research Animal Resources at the University of Minnesota Duluth. Squirrels were kept at 23°C with a 12∶12 light:dark schedule and fed Laboratory Rodent Diet 5001 (PMI Nutrition International). The sex of the squirrels used in each experiment is shown in Table S1.

Squirrels that were not designated to receive any adenovirus injections were placed on an ambient temperature and light cycle schedule resembling seasonal conditions in the wild. At the start of November animals were placed in 24 hour dark, food was removed and the ambient temperature was reduced to 4°C where it remained until mid-March when the temperature was increased to 23°C, light cycle was returned to 12∶12 light:dark, and food was available *ad libitum*. All squirrels had access to water during the entire year.

### Transmitter implant

Squirrels were fully anesthetized with isoflurane (Phoenix Pharmaceutical, Inc.) for all surgical procedures. The surgical site was prepared and disinfected with betadine (Purdue Products L.P., povidone-iodine, 7.5%) and 70% ethanol. A CTA-F40 transmitter (Data Sciences International) capable of collecting body temperature, electrocardiogram (ECG), and activity data was sterilized by soaking in Actril Cold Sterilant (Minntech) and placed inside the abdominal cavity ventral to the intestines. The ECG leads of the transmitter were tunneled subcutaneously and secured so the exposed sections of the two ECG leads created a diagonal line crossing the heart. The transmitter was secured and incisions were closed using sutures or surgical staples (3 M, Precise©, DS-25). Anesthesia was ceased and the squirrel was monitored for anesthetic recovery. All animals were given 15 mg/kg ibuprofen in their water (Children's Motrin, ibuprofen 100 mg per 5 ml) and fed freely during recovery from transmitter implant for at least 7 days.

### Isolation of ground squirrel FGF21 cDNA

The FGF21 cDNA sequences from multiple species were aligned and primers that flanked a conserved region were selected. The primers selected were: primer A) 5′ AAG CCC ACC TGG AGA TCA GG 3′ (20 mer) and primer B) 5′ TCT GAA ACT GCA GGC CTC AGG 3′ (21 mer). These primers flanked an approximately 200 bp region of cDNA. Low-stringency PCR was performed with the following conditions: 94°C 2 min; 40 cycles of 94°C 30 sec, 53°C 30 sec, 72°C 1 min; then 72°C 7 min; 4°C hold. This was followed by 5′ and 3′ Rapid Amplification of cDNA Ends (RACE). Primer B was used for 5′ RACE with the following conditions: 94°C 3 min; 40 cycles of 94°C 30 sec, 64°C 30 sec, 72°C 1 min; then 72°C 7 min; 4°C hold. Primer A was used for 3′ RACE with the following conditions: 94°C 3 min; 40 cycles of 94°C 30 sec, 60°C 30 sec, 72°C 1 min; then 72°C 7 min; 4°C hold.

### Development of gsFGF21-expressing adenovirus

**Figure 1:**
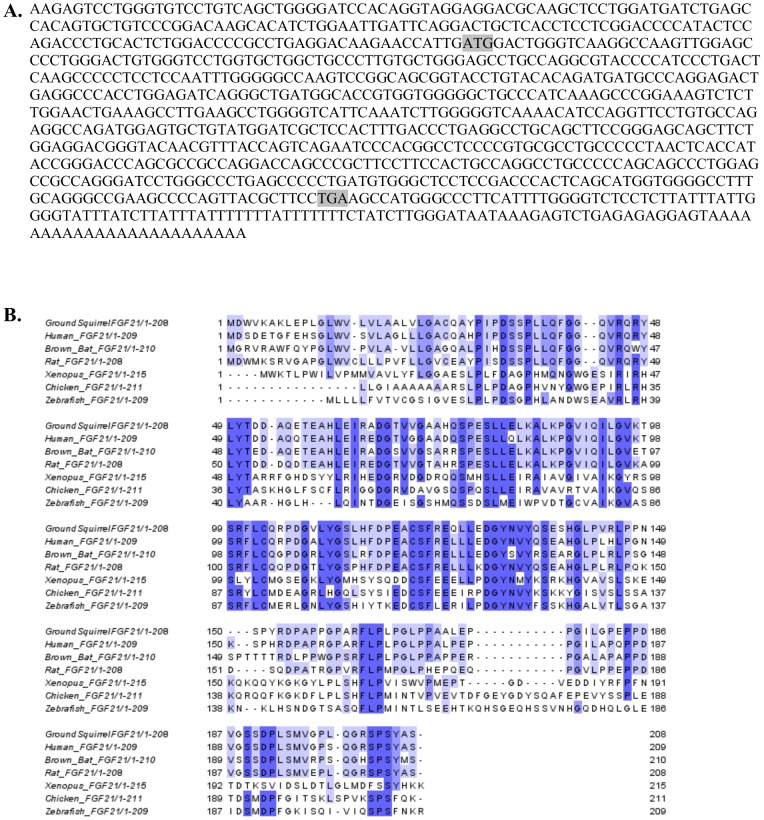
Thirteen-lined ground squirrel FGF21 cDNA and protein sequence. (**A**) Sequence of FGF21 cDNA isolated by RACE. The start and stop codons are highlighted in gray. (**B**) Translation of the above ground squirrel cDNA aligned with FGF21 protein sequences from multiple species using CLUSTALW (1.81). The sequences are colored according to percentage identity with >80% dark blue, >60% middle blue, >40% light blue, and < = 40% white.

The cre-loxP method was used to produce recombinant adenoviruses as described in Gerard and Meidell [25]. Briefly, the ground squirrel FGF21 cDNA sequence (645 bp total with 627 bp ORF; Figure 1A) was inserted into the *Xba*I and *Hin*dIII sites in the polylinker region of the pACCMVpLmP1 (−) loxP-SSP plasmid. The recombinant FGF21 virus (AdFGF21) and the control adenovirus that did not contain an insert (AdRR5) were cloned by plaque assay and propagated on 911 cells. For *in vivo* use, crude stocks were purified sequentially by centrifugation on CsCl step gradients and gel filtration on Sepharose CL-4B columns equilibrated with Tris-buffered isotonic saline (137 mM NaCl, 5 mM KCl, 10 mM Tris-HCl pH 7.4, 1 mM MgCl_2_). Absorbance at 260 nm (A_260_ of 1 equals 1×10^12^ particles/ml) was used to determine particle concentration. After the addition of 10% glycerol, viruses were stored frozen at −80°C at concentrations between 10^12^ and 10^13^ particles/ml until use.

### Treating primary adipocytes with FGF21-containing conditioned media

Primary pre-adipocytes were isolated from postnatal day (P) 4 wild type mice, grown to confluency in 6-well plates, and differentiated in media containing high-glucose DMEM plus 10% FBS, 5 μg/ml insulin, 0.5 mM isobutylmethylxanthine, and 1.0 μM dexamethasone [26]. After 2 days, differentiation was induced, media was replaced every other day for 6 more days with media containing high glucose DMEM plus 10% FBS and 5 μg/ml insulin. On day 10 of differentiation, media was replaced with serum-free high glucose DMEM for 24 hrs and adipocytes were treated with serum-free conditioned media from permissive 911 cells infected with adenovirus expressing either control, murine FGF21, or squirrel FGF21 for 10 minutes. Cells were collected in 300 μl lysis buffer containing 10 mM Tris-HCl, pH 7.4, 5 mM EDTA, 5 mM EGTA, 150 mM NaCl, 10% Glycerol, 1% NP-40, 0.5% Triton X-100, with complete protease inhibitor cocktail (Roche) and phosphatase inhibitor cocktail (Roche) per well. Samples were centrifuged at 13,000 rpm for 30 minutes at 4°C and supernatants were collected and diluted with 2× SDS sample buffer (Sigma), boiled for 3 minutes, and frozen at −20°C until processed. 50 μg of lysates were resolved on a 10% SDS-polyacrylamide gel and transferred to a nitrocellulose membrane (Bio-Rad). The membrane was blocked in 5% milk/TBS-T and probed with a specific P-ERK1/2 (thr202/tyr204) (Cell Signal) or total-ERK antibody (Cell Signal) at a 1∶1000 diultion at 4°C overnight followed by a secondary horseradish peroxidase-conjugated antibody. Proteins were resolved by Enhanced Chemiluminescense (ECL) western blotting substrate (Pierce) according to manufacturers' instructions. Primary antibodies were prepared in a 3% BSA/TBS-T solution and secondary antibodies in a 5% milk/TBS-T solution.

### Adenovirus infusion into ground squirrels

An anesthetized squirrel was weighed, animal length was measured, the left thigh and lower abdomen were shaved, and the squirrel was placed under a dissecting microscope (Bauch and Lomb, 1× to 2.5× magnification). A 1.5 cm incision was made through the epidermis at the anterior end of the thigh above the knee joint and excess adipose tissue was removed in order for observation of blood vessels. The characteristic bifurcation of the femoral vein and artery in the ground squirrel thigh was located. Silk suture was tied around the posterior end of the cleaned artery and a micro-hemostat was used to create tension. A piece of silk suture was tied around the anterior end of the cleaned femoral artery section. Micro-scissors (Miltex, 18–1618) were used to make a diagonal incision approximately half-way through the artery. A cannula was composed of 3 cm of PE10 tubing (Polyethylene, Braintree Scientific) with a beveled tip inserted into 30 cm of PE50 tubing (Polyethylene, Braintree Scientific) with a 23 gauge needle (Kendall Monoject, Polypropylene Hub) in the other end of the PE50 tubing. The cannula was filled with sterile saline containing 100 units USP per mL heparin sodium (AAP Pharmaceutical). The cannula was carefully inserted into the incision in the femoral artery without breaking the artery or puncturing the opposite side with the beveled cannula tip. While holding the cannula secure, and clamping gently to the artery to prevent blood loss, the anterior silk suture was loosened. The cannula was gently threaded into the artery taking care to keep the artery intact while feeling for the connection to the iliac artery and not forcing past that connection until approximately 2 cm of the PE10 tubing was inserted. The cannula was secured to the artery with silk suture. One ml of blood was removed through the cannula using a 1 ml syringe (Kendall Monoject Tuberculin Syringe, Without Needle). The squirrel and dissection microscope were moved to a Biosafety Level-2 Cabinet.

Adenoviral solution was prepared at a concentration of 7.5×10^12^ particles per kilogram squirrel weight by dilution in 0.9% sterile saline. This concentration is sufficient to transduce approximately half of all cells in mammalian liver. The diluted adenoviral solution was injected into the squirrel through the cannula using a 1 ml syringe followed by 1 ml of 0.9% sterile saline. The cannula was carefully removed from the femoral artery and the artery was securely closed. The hemostat was removed from the posterior silk suture and suture ends were trimmed. The incision was closed using surgical staples. The squirrel was given 15 mg/kg ibuprofen in the continuously available water bottle (Children's Motrin, ibuprofen 100 mg per 5 ml) and recovery from anesthesia was monitored.

### Monitor and collection of physiological parameters

Dataquest A.R.T. 4.1, gold edition software had been installed in the PC connected to the physiological monitoring equipment. Squirrels given control adenovirus (AdRR5) or adenovirus encoding ground squirrel FGF21 (AdFGF21) were allowed to recover from anesthesia before monitoring was initiated and continued for a minimum of 7 days. Analysis of the collected physiological data is presented as mean and minimum T_b_ and HR and the percent weight change over 7 days. The mean T_b_ and HR data provide a value for each animal. If a squirrel enters torpor these values can indicate the amount of time spent in the torpid versus active state, with lower mean values indicating more time spent in torpor over 7 days. The minimum T_b_ and HR indicate the depth of torpor for each squirrel and a lower minimum indicates a deeper torpor bout.

### Tissue and serum collection

At the end of the seven-day period the squirrel was anesthetized, weighed, and a rectal body temperature was taken. Blood was collected by cardiac puncture, stored on ice and allowed to clot. The sample was centrifuged and the serum was aliquoted and stored at −80°C until analysis. The squirrel organs, including brain, heart, liver, muscle, spleen, adrenals and white adipose tissue, were removed, trimmed, and snap frozen in liquid nitrogen in cryovials (Nunc Cryotube Vials, 1.8 ml). All tissues were stored in a freezer at −80°C or in liquid nitrogen as space allowed.

### Serum metabolite and insulin measurements

Serum β-hydroxybutyrate was measured using β-Hydroxybutyrate LiquiColor Kit (Stanbio Laboratory, 2440–058). Serum triglyceride (TG) concentrations were measured using an L-Type TG H kit (Wako). Serum glucose concentrations were measured using a Glucose LiquiColor kit (Stanbio Laboratories, 1070–125). Serum insulin was measured using an Ultra Sensitive Mouse Insulin ELISA Kit (Crystal Chem Inc, 90080). All assays were done as per kit directions.

### Gene expression assays

RNA extraction was completed using RiboPure Kit (Ambion) as per directions. The concentration of RNA was determined by measuring the absorbance of the sample at 260 nm, and the integrity was confirmed using the 260/280 nm absorbance ratio (Nanodrop). Preparation of cDNA from the extracted RNA was done using QuantiTect Reverse Transcription Kit (Qiagen) as per directions. Each cDNA was diluted to 5 ng/µl for use in PCR and stored at −80°C. Mock cDNA preparations with no reverse transcriptase were run through a quantitative PCR to detect any genomic DNA contamination.

Quantification of target gene RNA was done using the Rotor-Gene SYBR Green PCR Kit (Qiagen) as per the directions. The 2× Rotor-Gene SYBR Green Master Mix in the kit contained HotStar *Taq* Plus DNA Polymerase, Rotor-Gene SYBR Green PCR Buffer, and SYBR Green I. The template cDNA was thawed on ice and the PCR reagents and primers were thawed at room temperature. A master mix was made including 12.5 µl 2× Rotor-Gene SYBR Green Master Mix, 0.25 µl of each primer, and 10 µl of RNase-free water per reaction. A 23 µl aliquot of master mix and 2 µl of cDNA were added to each 0.1 ml Rotor-Gene PCR reaction tube. The tubes were capped and loaded in the RotorGene 3000 PCR instrument (Corbett Research). The reactions were incubated at 95°C for 5 min and cycled at 95°C for 5 sec and 60°C for 15 sec, for 35 cycles. Cyclophilin A was determined to be an acceptable control gene for comparison of target gene expression.

### Primer Design and Verification

To design primers for possible control genes and genes of interest, a gene sequence was searched in the BLAST database, the ENSEMBLE database, or from previously acquired sequences from the Andrews lab. The highest quality sequence available was used for primer design. Exons of the DNA sequences were determined by matching the thirteen-lined ground squirrel sequence to a reference sequence in the BLAST database. The primers were designed by using either OligoPerfect Designer (Invitrogen) or Primer Quest (IDT). The primer pairs, their melting temperatures, GC content, and amplicon size can be viewed in Table 1.

**Table 1:** Q-RT-PCR Primers.

Target Gene	Amplicon Size (bp)	Primer Sequence	GC Content (%)	Melt Temp (°C)
CycA	224	F': GTTGGATGGCAAGCATGTGGTCTT	50	60.00
		R': TGGGATATTGCGAGCAGATGGGAT	50	60.00
βKlotho	85	F': TTGGTGAAGCTCTGGATCAC	50	60.02
		R': CTGCCCTGTAGGTGTCGTTA	55	59.95
PTL	150	F': TGAACTGCATCTGTGTGGACTGGA	50	60.00
		R': TGACATGGACATTGGAAGGCGAGT	50	60.30
HMGCS	106	F': AAGGCCTCCCTTTACCTTTCCACA	50	60.10
		R': AGCCAAGTCCTGAGCAGAGTGATT	50	59.90
CPT1a	124	F': TTTGACCTGGAGAGGAACCCAGAA	50	59.50
		R': AGGAGACGTGGAAGTGGATGAAGT	50	59.40
HSL	166	F': AGATGAGAAGGCACTGGGCATGAT	50	60.10
		R': ACTGCGTCGCATTGACTCTACTGT	50	60.00

## Results

### Thirteen-lined Ground Squirrel FGF21 Sequence

The thirteen-lined ground squirrel FGF21 sequence was isolated by low stringency PCR followed by 3′ and 5′ RACE using primers derived from conserved FGF21 sequences seen in multiple species (Figure 1A). A BLASTn search produced a top hit of reference sequence NM_019113.2 [*Homo sapiens* fibroblast growth factor 21 (FGF21) mRNA] with 676/793 (85%) identities at the nucleotide level [27]. Translation of the mRNA sequence using ExPASy generated a protein sequence containing 208 amino acids (Figure 1B) with the highest BLASTp match to human FGF21 (gb|AAH18404.1) with an expect score of 3*e*-86, 178/209 (85%) amino acids, 187/209 (89%) positives, and 1/209 (0%) gaps [28]–[30]. The translated ground squirrel sequence was aligned to FGF21 sequences from *Homo sapiens* (UniProt ID Q9NSA1), *Myotis lucifigus* (little brown bat, UniProt ID G1P6U5), *Rattus norvegicus* (rat, UniProt ID Q8VI80), *Xenopus tropicalus* (western clawed frog, UniProt ID B7U4G3), *Gallus gallus* (chicken, UniProt ID F1NYA6), and *Danio rario* (zebrafish, UniProt ID F1QR52) using CLUSTAL W on the UniProt database ([31]; Figure 1B). Based on this analysis the RACE-isolated cDNA sequence was concluded to encode thirteen-lined ground squirrel FGF21.

### Expression of FGF21 in ground squirrels

**Figure 2:**
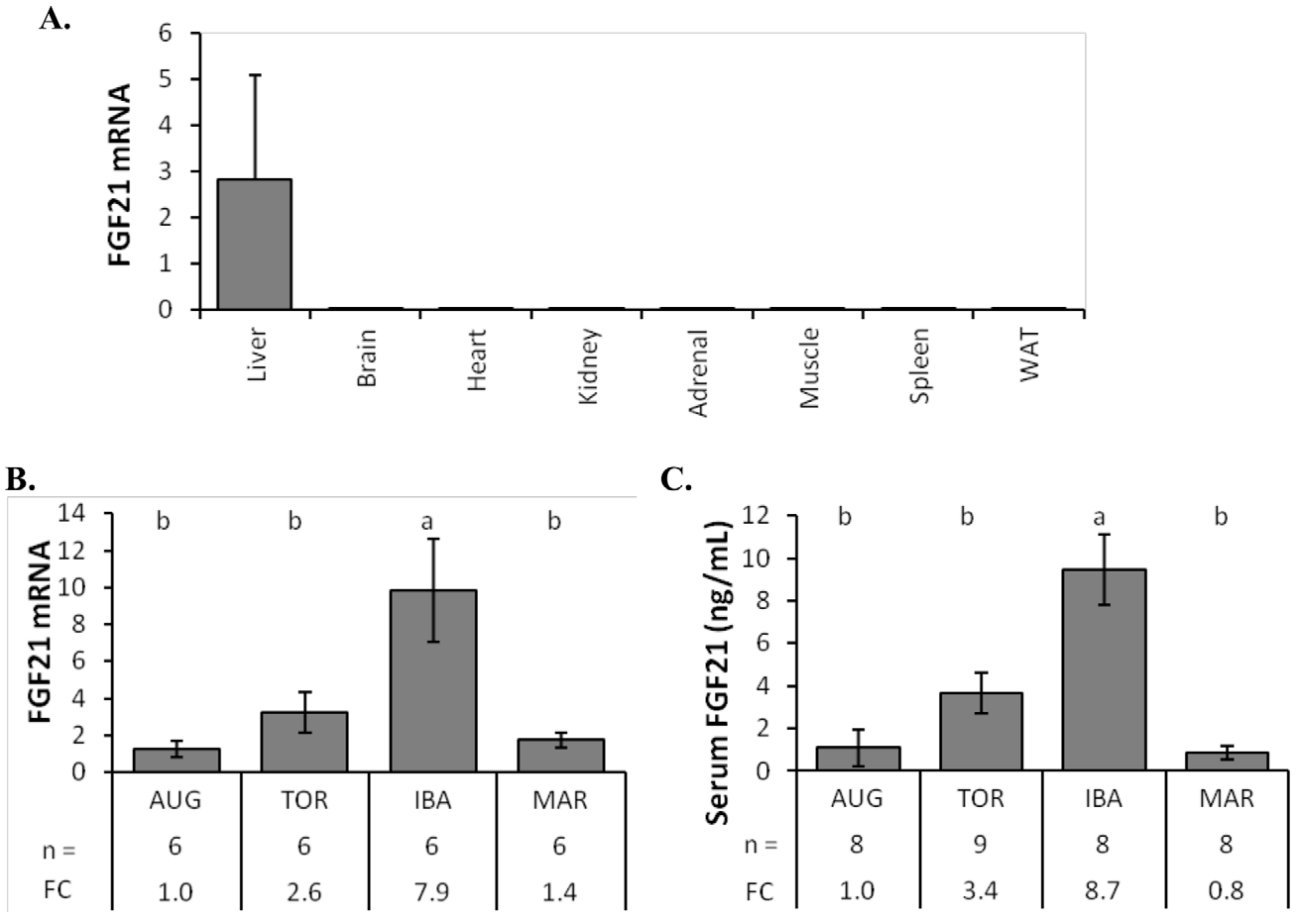
FGF21 expression in thirteen-lined ground squirrels. (**A**) FGF21 mRNA was measured by qRT-PCR relative to 18S RNA levels in multiple tissues of the thirteen-lined ground squirrel. RNA from 6 squirrels with 2 each from August active, winter torpid, and winter IBA were used. (**B**) Relative liver FGF21 mRNA levels during indicated activity states. (**C**) Concentration of serum FGF21 protein during indicated activity states. In panels B and C, fold change are shown relative to August active levels and data bars that do not share the same letter above the bar are significantly different from each other using ANOVA followed by Tukey's HSD (*p*<0.01). Errors bars show standard error of the mean. *Abbreviations* – n, number of animals; FC, fold change; AUG, August active; TOR, torpor; IBA, interbout arousal; MAR, March active; WAT, white adipose tissue.

The presence of endogenous FGF21 in various ground squirrel tissues was measured by quantitative real-time PCR (qRT-PCR) of RNA samples from liver, brain, heart, kidney, adrenal, skeletal muscle, spleen, and white adipose tissue (WAT) from 6 different animals. The 6 animals were comprised of two squirrels each from three seasonal time points: August active, winter torpid, and winter interbout arousal (IBA). Hibernating squirrels regularly cycle through torpor bouts that are interrupted by brief IBAs approximately every 10 days. The expression of FGF21 was found to be exclusive to liver tissue with a mean relative expression level of 2.8 in liver and less than 0.005 in all other tissues examined (Figure 2A). Expression of the FGF21 gene in mice is also predominantly found in the liver [11]. The absence of FGF21 mRNA in thirteen-lined ground squirrel heart, skeletal muscle, and white adipose tissue throughout the year was also confirmed by deep sequencing the transcriptome of these tissues using the Roche 454 platform [32]. FGF21 mRNA was also absent from brown adipose tissue (BAT), brain cortex and hypothalamus as determined by Illumina sequencing of the respective transcriptomes at four different times/activity states: April active, October active, torpor and IBA (data not shown).

After determining tissue specificity, FGF21 mRNA and protein levels were measured using samples collected from four different activity states seen in the seasonal hibernator: summer active, torpor, interbout arousal, and spring arousal. Summer active squirrels (August) are in the process of consuming excess food to accumulate and store lipid in adipose tissue in preparation for the upcoming hibernation season. Torpid and IBA squirrels are in the middle of the hibernation season (January and February) and have not consumed food since the end of October. March active squirrels have aroused from the hibernation season, have been active and eating for approximately 2 weeks, and are prepared to reproduce as indicated by the descent of testes and enlargement of vulvae. FGF21 mRNA levels in ground squirrel livers were measured in 6 animals at each of the four activity states by qRT-PCR (Figure 2B). Statistical analysis using ANOVA (F = 8.9) followed by Tukey's HSD (*p*<0.01) showed that the level of FGF21 mRNA level was significantly higher during IBAs than any other activity state and 7.9-fold higher than August active squirrels. The normothermic IBAs provide an opportunity for transcription and synthesis of proteins needed during the following hypothermic torpor bouts [33], [34].

FGF21 protein is produced in the liver and secreted into the blood. Serum samples were prepared from whole blood and used for FGF21 protein measurements by enzyme-linked immunosorbent assay (ELISA). Consistent with liver mRNA data, the FGF21 protein concentrations in August active, winter torpid, winter IBA, and March active squirrel serum samples were 1.09, 3.64, 9.45, and 0.86 ng/ml respectively (Figure 2C). Protein concentrations were compared by ANOVA (F = 13.8) followed by Tukey's HSD and showed a significantly higher serum concentration of FGF21 during IBA than other states, and 8.7-fold higher than in August active squirrels. The winter torpid animals also had a higher level of serum FGF21 compared to active animals from March or August, but the differences were not statistically significant. Circulating FGF21 protein concentrations were therefore highest in IBA squirrels preceding another round of torpor, consistent with the hypothesis that FGF21 signaling is involved in torpor induction during hibernation.

### Presence of FGF21 co-factors

**Figure 3:**
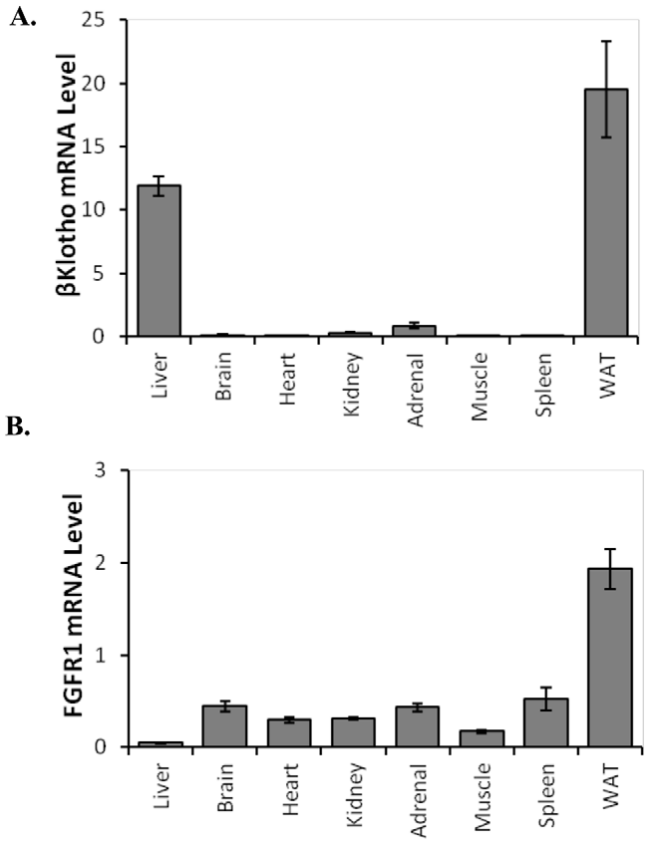
Relative levels of FGF21 signaling component mRNAs in ground squirrel tissues. The relative levels of mRNA were measured in eight tissues from thirteen-lined ground squirrels with 2 squirrels in August active state, 2 squirrels in winter torpid state, and 2 squirrels in IBA state for a total of 6 squirrels. (**A**) βKlotho mRNA was detected predominantly in liver and white adipose tissue (WAT). (**B**) FGFR1 mRNA was detected in all tissues at a low level with the highest expression in WAT. Error bars show standard error of the mean.

FGF21 interacts with cell surface receptor heteromers composed of FGF receptors in complex with the transmembrane protein, βKlotho [14]–[18]. In the thirteen-lined ground squirrel, βKlotho mRNA was present in both liver and WAT (Figure 3A) as shown by qRT-PCR with relative expression levels of 11.9 and 19.5, respectively, and in BAT as shown by Illumina transcriptome sequencing (data not shown). Relative levels of βKlotho were less than 1 in brain, heart, kidney, adrenal, skeletal muscle, and spleen. The presence of βKlotho mRNA in WAT and lack of βKlotho mRNA in heart and skeletal muscle were also confirmed by transcriptome analysis [32]. βKlotho mRNA levels in liver from August active squirrels was higher compared to March active squirrels (Figure S1A). In WAT βKlotho levels did not differ with activity state (Figure S1B). FGF receptor 1 (FGFR1) expression was highest in WAT, with lower levels detected in other tissues by qRT-PCR (Figure 3B) and in BAT as shown by Illumina transcriptome sequencing (data not shown). This suggests that FGF21 is capable of βKlotho-mediated signaling activity in liver, WAT and BAT, but not the other tissues tested.

### Thirteen-lined ground squirrel FGF21 signaling capability

**Figure 4:**
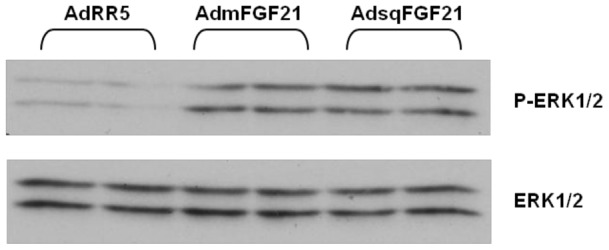
Adenoviral-mediated expression of cloned thirteen-lined ground squirrel FGF21 is capable of signaling in mouse adipocytes. Following FGF21 activation of FGFR, the MAPK pathway is initiated involving phosphorylation of ERK 1 and 2. The phosphorylation of ERK1/2 (P-ERK1/2) resulting from thirteen-lined ground squirrel FGF21 (AdsqFGF21) is similar to the positive control using mouse FGF21 (AdmFGF21). Adenovirus not encoding FGF21 (AdRR5) was used as a control. Each adenoviral assay was done in duplicate.

The thirteen-lined ground squirrel FGF21 cDNA (Figure 1A) was cloned into an adenoviral expression vector so that we could elevate levels of the hormone *in vivo* and test our hypothesis that FGF21 signaling is involved in torpor induction during hibernation. To confirm that the adenovirus-expressed protein was functional, primary mouse adipocytes were used to determine whether the ground squirrel FGF21could induce ERK phosphorylation *in vivo* (Figure 4). Adipocytes were treated with conditioned media from 911 cells infected with adenovirus containing no insert, adenovirus with an insert encoding mouse FGF21, or adenovirus containing an insert encoding the thirteen-lined ground squirrel FGF21. Proteins were isolated from treated cells and ERK phosphorylation was examined by western blotting. Similar to the mouse protein, thirteen-lined ground squirrel FGF21 induced ERK phosphorylation demonstrating that the protein is functional.

### Adeonoviral-mediated increase of FGF21 in thirteen-lined ground squirrels

FGF21 levels were artificially increased in thirteen-lined ground squirrels to determine whether higher concentrations of FGF21 would induce a hibernation-like torpor. Circulating FGF21 concentrations were elevated by infusing animals with an adenovirus encoding the *I. tridecemlineatus* FGF21 protein (AdFGF21). As a control, squirrels were also infused with the same adenoviral vector not containing an insert (AdRR5). The physiological response of ground squirrels was evaluated using surgically implanted transmitters that continuously monitored body temperature, heart rate (ECG), and movement.

To measure the ability of elevated FGF21 to induce various aspects of the hibernation phenotype, experiments were conducted over a 7 day period at different months of the year under various environmental conditions of food availability (fed or fasted), ambient temperature (°C), and light:dark (l∶d) cycles. Three different experiments were performed to examine the effect of elevated FGF21 under conditions that are conducive to torpor: 1.) March/April – fasted, 5°C, 24 h d; 2.) August – fasted, 5°C, 24 h d; 3.) October – fasted, 5°C, 24 h d. We also conducted one experiment where conditions are conducive to normal animal activity: April – fed, 23°C, 12 h∶12 h l∶d. The physiological parameters measured are presented as mean body temperature (T_b_) and heart rate (HR), minimum T_b_ and HR, and percent change in weight over the 7-day period.

**Figure 5:**
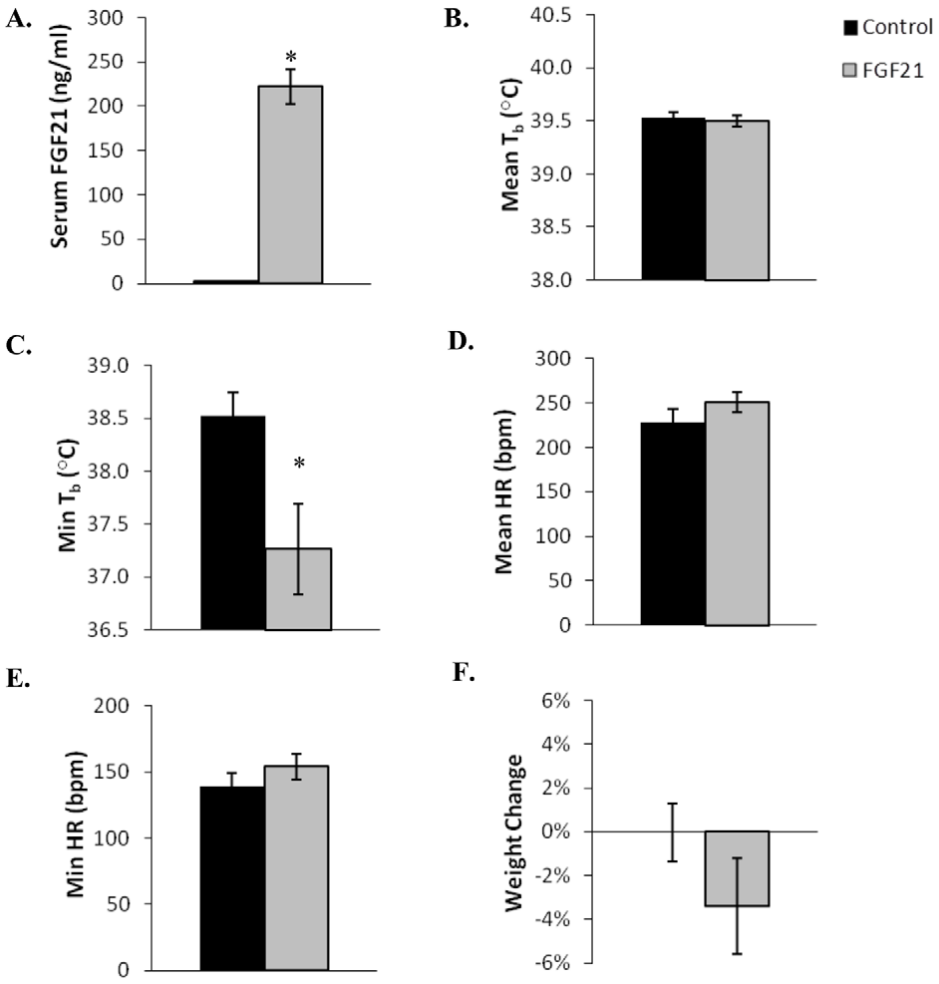
Physiological parameters in fed April thirteen-lined ground squirrels infused with AdFGF21 (n = 8) and control adenovirus AdRR5 (n = 8). The squirrels were kept at 23°C with a 12∶12 light:dark cycle with food *ad libitum* for 7 days. Serum concentration of FGF21 and changes in body weight were measured 7 days after infusion with either AdFGF21 (FGF21) or AdRR5 (Control) adenoviruses. Body temperatures and heart rate were measured continuously over 7 days using implanted transmitters. Statistical significance (*) was evaluated by Student's t-test. (**A**) Serum concentration of FGF21 (*, *p*<0.0001). (**B**) Mean body temperatures. (**C**) Mean minimum body temperature (*, *p* = 0.012). (**D**) Mean heart rate (HR). (**E**) Mean minimum heart rate. (**F**) Change in weight. Error bars show standard error of the mean.

**Figure 6:**
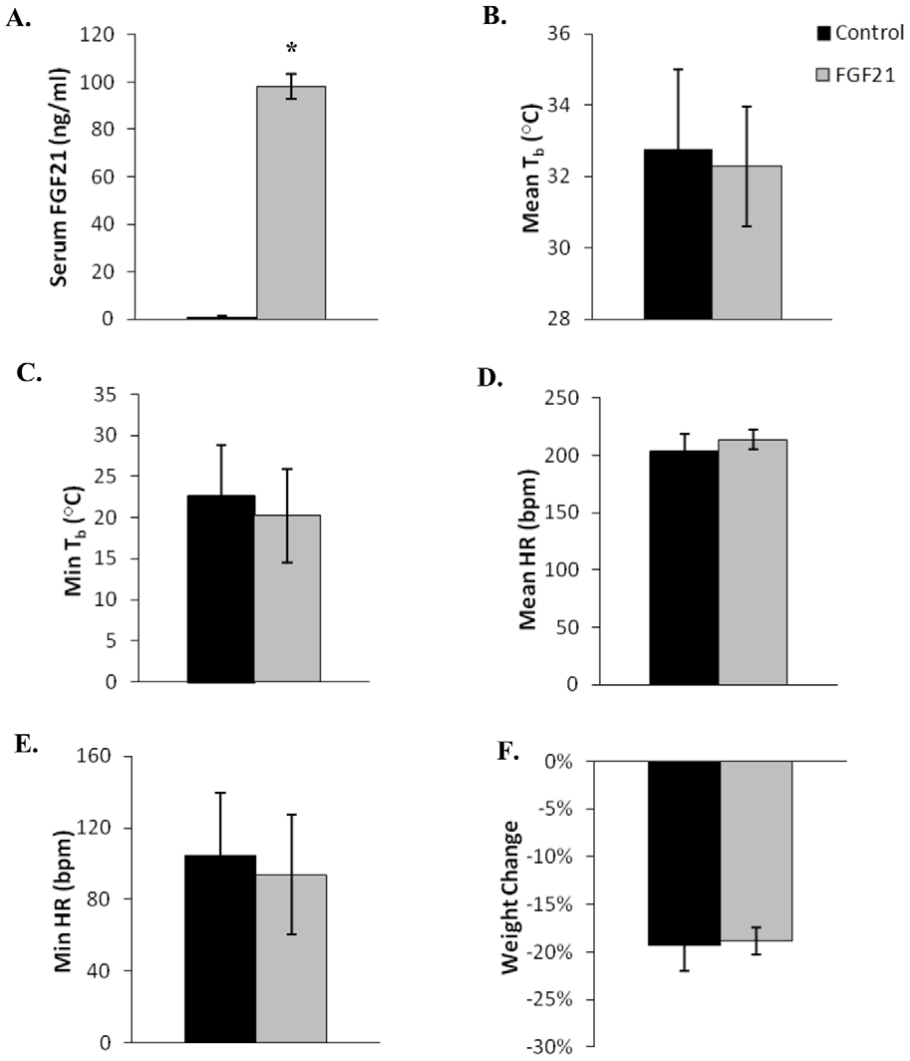
Physiological parameters in fasted March-April thirteen-lined ground squirrels infused with AdFGF21 (n = 8) and control adenovirus AdRR5 (n = 7). The squirrels were kept at 5°C, in 24 h darkness, without food for 7 days. Serum concentration of FGF21 and changes in body weight were measured 7 days after infusion with either AdFGF21 (FGF21) or AdRR5 (Control). Body temperatures and heart rate were measured continuously over 7 days using implanted transmitters. Statistical significance (*) was evaluated by Student's t-test. (**A**) Serum concentration of FGF21 (*, *p*<0.0001). (**B**) Mean body temperatures. (**C**) Mean minimum body temperature. (**D**) Mean heart rate (HR). (**E**) Mean minimum heart rate. (**F**) Change in weight. Error bars show standard error of the mean.

**Figure 7:**
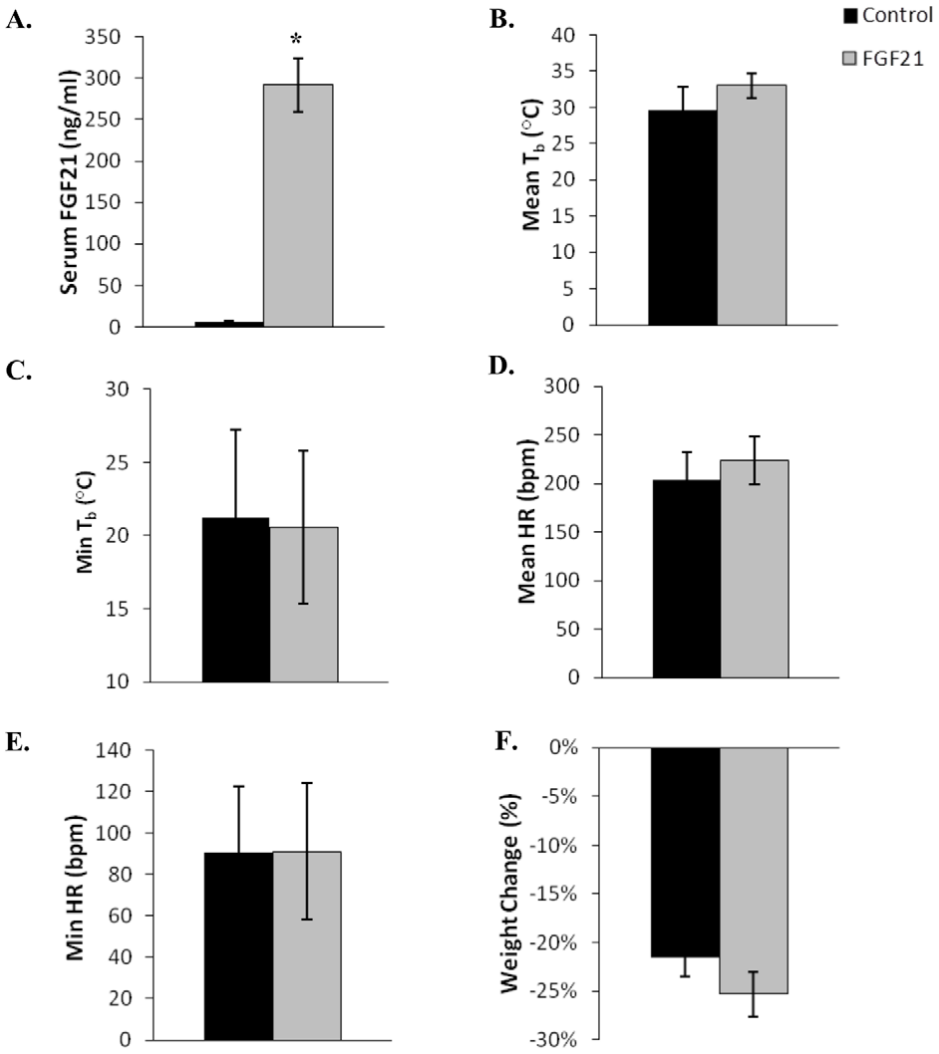
Physiological parameters in fasted August thirteen-lined ground squirrels infused with AdFGF21 (n = 8) and control adenovirus AdRR5 (n = 7). The squirrels were kept at 5°C, in 24 h darkness, without food for 7 days. Serum concentration of FGF21 and changes in body weight were measured 7 days after infusion with either AdFGF21 (FGF21) or AdRR5 (Control). Body temperatures and heart rate were measured continuously over 7 days using implanted transmitters. Statistical significance (*) was evaluated by Student's t-test. (**A**) **S**erum concentration of FGF21 (*, *p*<0.0001). (**B**) Mean body temperatures. (**C**) Mean minimum body temperature. (**D**) Mean heart rate (HR). (E) Mean minimum heart rate. (**F**) Change in weight. Error bars show standard error of the mean.

**Figure 8:**
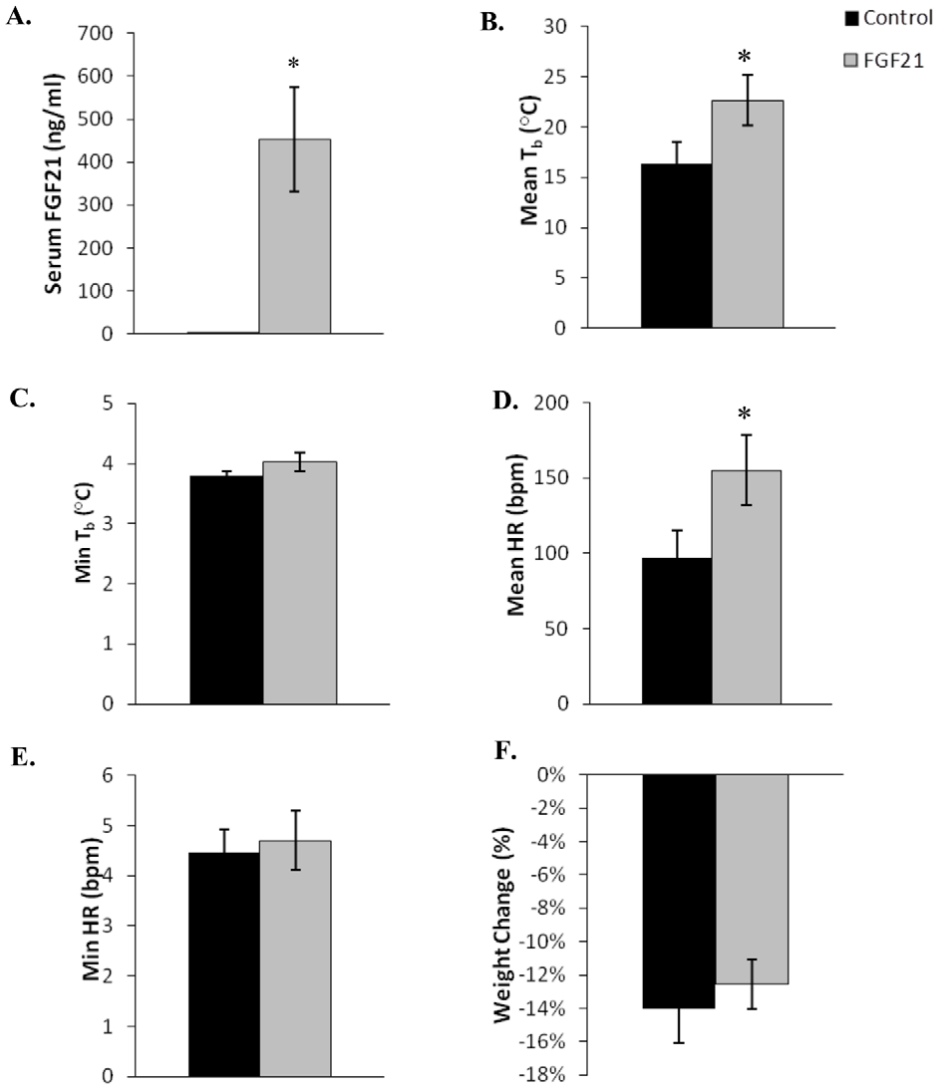
Physiological parameters in fasted October thirteen-lined ground squirrels infused with AdFGF21 (n = 8) and control adenovirus AdRR5 (n = 7). The squirrels were kept at 5°C, in 24 h darkness, without food for 7 days. Serum concentration of FGF21 and changes in body weight were measured 7 days after infusion with either AdFGF21 (FGF21) or AdRR5 (Control). Body temperatures and heart rate were measured continuously over 7 days using implanted transmitters. Statistical significance (*) was evaluated by Student's t-test. (**A**) Serum concentration of FGF21 (*, *p* = 0.004). (**B**) Mean body temperatures (*, *p*<0.05). (**C**) Mean minimum body temperature. (**D**) Mean heart rate (*, *p*<0.05). (**E**) Mean minimum heart rate. (**F**) Change in weight. Error bars show standard error of the mean.

Serum samples were prepared 7 days after adenoviral infusion to determine the concentration of FGF21 and the metabolic parameters shown in Table 2. Infusion of AdFGF21 significantly increased the concentration of serum FGF21 under all four sets of experimental conditions when compared to serum from AdRR5-infused squirrels (panel A of Figures 5–8). These measurements showed that AdFGF21 was an effective means of increasing FGF21 serum protein in wild-caught thirteen-lined ground squirrels.

**Table 2:** Serum Metabolites in Thirteen-lined Ground Squirrels with Increased FGF21 at 7 days following infusion (Values followed by SEM).

Experimental Conditions	Virus Type	n	Glucose (mM)		BHB (mM)		TG (mM)		FA (mM)		Insulin (ng/ml)	
Apr, fed, 23°C, 12∶12 l:d	Control	8	9.0	±0.76	0.2	±0.02	4.0	±0.72	0.15	±0.04	1.0	±0.17
	FGF21	8	9.1	±1.25	0.2	±0.01	4.3	±0.63	*0.06	±0.02	(n = 7) *0.6	±0.14
Mar/Apr, fast, 5°C, 24d	Control	4	(n = 5) 6.9	±0.62	2.1	±0.97	2.2	±0.59	0.72	±0.31	1.0	±0.26
	FGF21	5	6.6	±1.54	2.2	±0.85	1.4	±0.23	0.38	±0.12	1.4	±0.49
Aug, fast, 5°C, 24d	Control	7	5.9	±0.55	1.2	±0.39	5.0	±0.67	0.61	±0.17	(n = 6) 0.4	±0.27
	FGF21	8	7.2	±0.98	1.2	±0.32	4.9	±0.98	0.56	±0.09	0.6	±0.24
Oct, fast, 5°C, 24d	Control	8	(n = 9) 7.2	±1.30	5.3	±1.05	4.1	±0.94	1.22	±0.14	(n = 7) 0.7	±0.71
	FGF21	8	9.6	±1.23	3.0	±1.11	5.2	±1.03	0.98	±0.20	0.4	±0.12

*Abbreviations* – Apr, April; Aug, August; Mar, March; Oct, October; BHB, D-beta-hydroxybutyrate; FA, fatty acids; TG, triglycerides.

### Active conditions – April fed

Within one month of spring arousal active thirteen-lined ground squirrels were infused with either AdFGF21 (n = 8) or control virus AdRR5 (n = 8). Serum concentration of FGF21 in control squirrels was 2 ng/ml versus 222 ng/ml in squirrels given AdFGF21, equaling a 111-fold increase in circulating FGF21 (*p*<0.0001, Fig. 5A). The squirrels were kept at 23°C, with a 12∶12 light:dark cycle with food and water *ad libitum* for 7 days. FGF21 overexpression significantly decreased serum insulin and free fatty acid concentrations but had no effect on serum glucose, triglyceride or BHB levels (Table 2). Data for the measured physiological parameters for April fed animals are shown in Figure 5B–F. There was no significant difference between the mean body temperatures of FGF21 and control squirrels (Figure 5B), but the mean minimum body temperature of 37.3°C in squirrels with elevated FGF21 was significantly lower than the 38.5°C (*p* = 0.012, Fig. 5C) seen in squirrels infused with control virus. No significant differences in mean heart rate or body weight changes were seen between animals infused with AdFGF21 or the control AdRR5 virus. No squirrels entered torpor from either experimental group.

### Torpor conditions – March/April fasted

Near the end of March and in early April, 8 squirrels were infused with AdFGF21 and 7 squirrels were infused with AdRR5 followed by torpor-promoting conditions of 5°C, 24 hours dark, water *ad libitum*, but no food. The serum FGF21 concentration of control squirrels was 0.82 ng/ml, whereas squirrels infused with AdFGF21 had a significantly higher concentration of 98.10 ng/ml – representing a 119-fold increase (*p*<0.0001, Fig. 6A). The mean minimum body temperature was 22.6°C for control squirrels and 20.2°C for the squirrels with increased FGF21. Of the 15 squirrels approximately half entered torpor – 4 given AdFGF21 and 3 given AdRR5. As expected, there was a significant difference in weight loss between the squirrels that entered torpor (17%) and those that did not enter torpor (24%) regardless of the adenovirus administered (*p* = 0.0027). However there were no significant differences in serum metabolite parameters, body temperatures, heart rates, or weight changes between the control squirrels and squirrels with increased FGF21 (Table 2 and Figure 6).

### Torpor conditions – August fasted

In August, 8 squirrels received AdFGF21 and 7 squirrels received AdRR5. The squirrels were maintained and monitored in 24 h darkness for 7 days at 5°C with no food. Control squirrels infused with AdRR5 had a serum FGF21 concentration of 5.62 ng/ml, while squirrels that received the AdFGF21 had a significantly higher concentration of 291.92 ng/ml, a 52-fold increase (*p*<0.0001, Fig. 7A). Of the 15 squirrels, 3 control squirrels entered torpor and 4 squirrels with elevated FGF21 entered torpor. Of the torpid animals the duration of body temperature changes that indicated entrance, maintenance, and arousal phases of torpor were not different between FGF21-elevated and control animals. Overall, elevated levels of FGF21 did not affect weight loss in fasted thirteen-lined ground squirrels during August, but as expected there was significantly less weight loss in squirrels that entered torpor compared to squirrels that remained active under these fasting conditions. There were no significant differences in serum metabolites, body temperature, heart rate, or weight changes in control squirrels versus squirrels with increased FGF21 in August (Table 2 and Figure 7).

### Torpor conditions – October fasted

In October squirrels have acquired excess white adipose tissue and are ready to begin hibernation. The previous experimental conditions to promote torpor were repeated with 8 thirteen-lined ground squirrels receiving AdFGF21 and 7 squirrels receiving AdRR5. All squirrels were maintained without food at 5°C, 24 hours darkness and water *ad libitum* for 7 days. Control squirrels receiving AdRR5 had a serum FGF21 concentration of 4.25 ng/ml, where squirrels that received AdFGF21 had a significantly higher concentration of 452.68 ng/ml, or a 106-fold increase (*p* = 0.004, Fig. 8A). All 15 thirteen-lined ground squirrels entered torpor regardless of the adenoviral construct they received. The lengths of entrance, maintenance, and arousal phases of torpor did not differ significantly for squirrels with elevated levels of FGF21 compared to control squirrels. The mean 7-day body temperature and mean 7-day heart rate were significantly higher in squirrels with increased FGF21 levels, but other parameters measured did not differ between groups (Table 2 and Figure 8).

### Gene expression response to increased FGF21

The effect of increased FGF21 on gene expression was evaluated using qRT-PCR of RNA extracted from liver and WAT at the conclusion of each of the 7-day experiments. The mRNA levels of βKlotho, pancreatic triacylglycerol lipase (PTL), hydroxy-methylglutaryl-CoA synthase (HMGCS), carnitine palmitoyl transferase isoform 1A (CPT1a) and hormone sensitive lipase (HSL) were measured in liver tissue; and βKlotho, HSL and PTL were measured in WAT. Of the genes that FGF21 is known to activate in mice [13], PTL is of primary interest because it is expressed at high levels in WAT during hibernation and shows lipolytic activity at near-freezing temperatures [35]–[37].

βKlotho was detected in all experimental groups shown in Figures 5–8 indicating that increased FGF21 could signal downstream regulation of target genes in the liver and WAT of thirteen-lined ground squirrels, but elevated FGF21 had no effect on βKlotho expression in either tissue (Figure S1). The only significant difference in mRNA levels attributed to elevated FGF21 was a lower level of liver CPT1a mRNA in October squirrels (data not shown). There were no changes observed in HMGCS and HSL mRNA levels in the liver of squirrels containing increased FGF21, and no changes observed in HSL and PTL mRNA levels in WAT (data not shown).

## Discussion

We studied FGF21 function in a model hibernating mammal, the thirteen-lined ground squirrel (*Ictidomys tridecemlineatus*). This species naturally fasts from late fall until early spring and does not consume food during regular interbout arousals (IBAs). Wild-caught ground squirrels placed in hibernating conditions during the late fall show higher levels of serum FGF21 during torpor and a significant increase during IBAs (Figure 2C). IBAs provide a brief normothermic period (<24 h) when macromolecular reactions such as transcription and translation can occur [33], [34]. We hypothesized that FGF21 is expressed in the ground squirrel liver and released into the blood where it circulates throughout the body initiating metabolic rate reduction, fat catabolism and torpor. To test this hypothesis we increased the circulating level of FGF21 at various times of the year by adenoviral expression of the ground squirrel FGF21 sequence. This study is the first time that overexpression of a recombinant protein has been used in a wild hibernator to examine mechanistic aspects of the hibernation phenotype.

Hibernation is a seasonal phenomenon where body weight often doubles, as excess adipose tissue is required to survive the 5–6 months of fasting through the winter. In contrast, fasting-induced torpor in mice occurs when body mass is low and the main energy source is ingested food rather than stored body fat [38]–[40]. Reductions in mouse body temperature during the initial stages of fasting is independent of FGF21, but ketogenic diet-induced body temperature reductions are decreased in FGF21-knockout mice suggesting that FGF21 may be involved in lowering body temperature during long-term fasting [41]. Overexpression of FGF21 sensitizes mice to starvation-induced torpor, including decreases in both body temperature and physical activity [13]. We acknowledge that these mouse experiments using either transgenic or adenoviral-mediated strategies involved chronic exposure to pharmacologic levels of FGF21 [13]. Nevertheless, they led us to investigate whether FGF21 affects natural hibernation.

Here we show that recombinant thirteen-lined ground squirrel FGF21 elicits the expected signaling activity in tissue culture cells (Figure 4), but increasing serum levels of FGF21 in the hibernator is not sufficient to cause torpor (Figures 5–8). Despite a massive increase in circulating FGF21 following infusion with adenovirus encoding the ground squirrel protein (Figure 5A), fed thirteen-lined ground squirrels in April did not show any significant differences in heart rate or weight change during 7 days of continuous monitoring. April squirrels recently completed the previous hibernation season and are typically not prepared to become torpid, but rather are prepared to reproduce. Squirrels with increased FGF21 however did show decreased serum insulin and fatty acids. None of the April fed ground squirrels kept at 23°C with a 12∶12 light cycle entered torpor, but they did show a slight but significantly lower mean minimum body temperature with raised levels of FGF21.

Under conditions of constant dark, 5°C and no food, approximately half of the FGF21-enhanced and control thirteen-lined ground squirrels entered torpor in March/April and in August. However animals with elevated FGF21 did not enter torpor more frequently, nor was there a difference in any of the measured parameters of torpor, compared to animals infused with control virus. As expected, under October fasting conditions all thirteen-lined ground squirrels entered torpor and the measured parameters were very similar in animals with elevated FGF21 relative to control animals.

The normal increase in serum levels of FGF21 during torpor and IBA suggests an important role for this protein during hibernation. One possibility is that FGF21 is involved in lipid metabolism in the liver. Removal of FGF21 in knockout mice results in increased liver weight and excess liver lipids in response to a ketogenic diet [42], [43]. Thirteen-lined ground squirrels have noticeable changes in liver size and color during the hibernation season possibly due to changes in lipid content (unpublished observation). Recently, cold-exposed FGF21-knockout mice showed lower body temperatures than control animals following a 3-day exposure at 5°C, indicating a role for FGF21 in chronic adaptive thermogenesis [44]. Similarly, naturally occurring increases in circulating FGF21 during IBAs (Figure 2C) may be involved in thermogenesis during arousal from torpor.

The selective pressure of winter necessitates hibernation to assure year-to-year survival of several small rodent species. The importance of hibernation for animal survival underscores the tremendous evolutionary selection of this annual adaptation and demands regulatory mechanisms that cannot be easily compromised. This study shows that FGF21 is strongly regulated during torpor and IBA, but FGF21 overexpression in thirteen-lined ground squirrels is not sufficient to cause torpor in this naturally hibernating mammal.

## Supporting Information

Figure S1 **βKlotho expression in thirteen-lined ground squirrels.** βKlotho mRNA was measured by qRT-PCR relative to 18S RNA levels in (**A**) liver and (**B**) white adipose tissue of thirteen-lined ground squirrels during indicated activity states. Liver βKlotho mRNA was significantly higher in August active squirrels than March active squirrels, but did not change in WAT. βKlotho mRNA was measured by qRT-PCR relative to cyclophilin A mRNA in (**C**) liver and (**D**) WAT of squirrels that received AdRR5 (black bars) or AdFGF21 (gray bars) for the indicated experiments corresponding to Figures 5–8. For all panels, data bars that do not share the same letter above the bar are significantly different from each other using ANOVA followed by Tukey's HSD (*p*<0.01). Errors bars show standard error of the mean. *Abbreviations* – n, number of animals; AUG, August active; TOR, torpor; IBA, interbout arousal; MAR, March active; WAT, white adipose tissue.
(TIF)

Table S1 **Sex of squirrels used in each experiment.**
(DOCX)
