# Supplementary figures and images for: Metabolic Hormone FGF21 Is Induced in Ground Squirrels during Hibernation but Its Overexpression Is Not Sufficient to Cause Torpor
Source: PLoS One. 2013 Jan 2;8(1):e53574. doi: 10.1371/journal.pone.0053574 (PMC3534659; doi:10.1371/journal.pone.0053574)

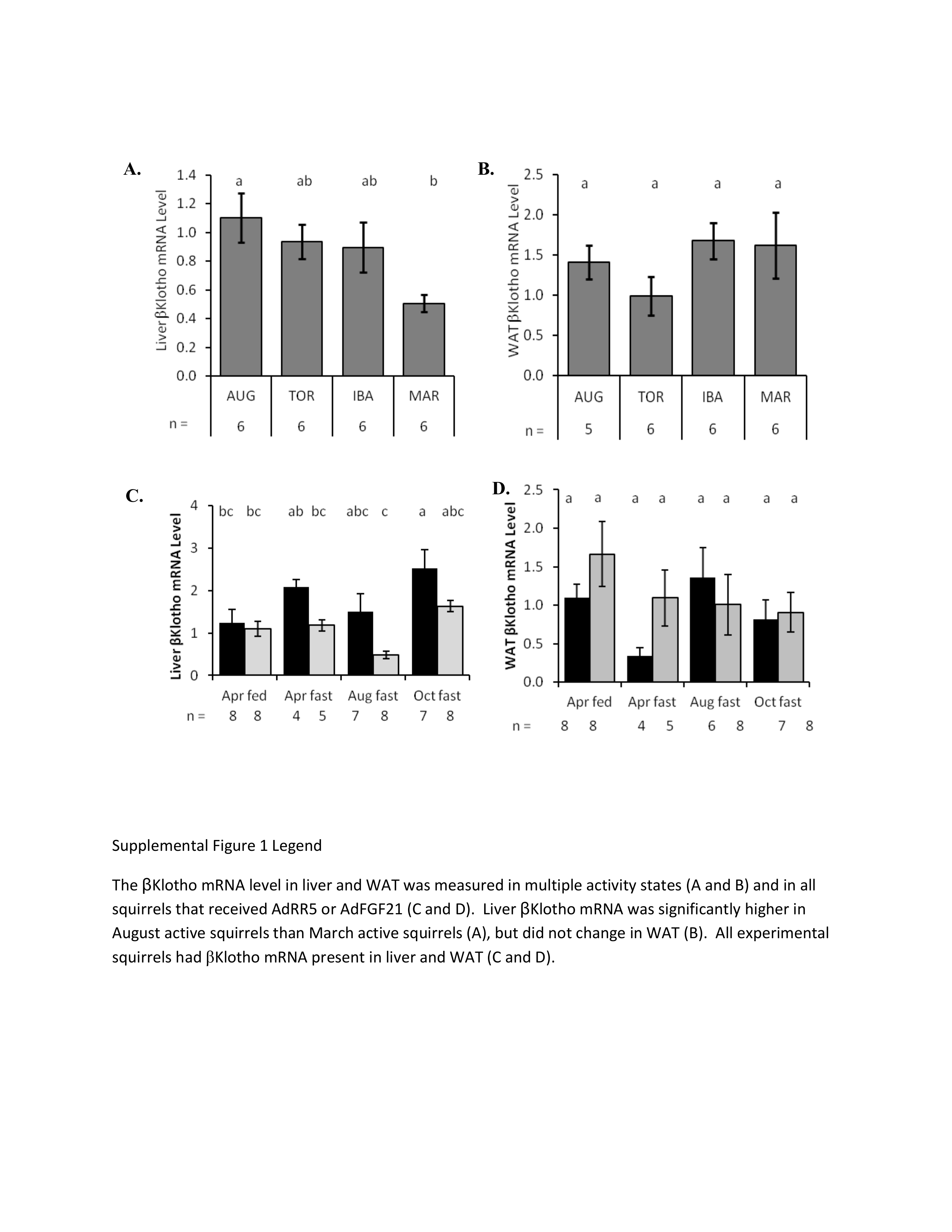

Supplement: Figure S1 — βKlotho expression in thirteen-lined ground squirrels. βKlotho mRNA was measured by qRT-PCR relative to 18S RNA levels in (A) liver and (B) white adipose tissue of thirteen-lined ground squirrels during indicated activity states. Liver βKlotho mRNA was significantly higher in August active squirrels than March active squirrels, but did not change in WAT. βKlotho mRNA was measured by qRT-PCR relative to cyclophilin A mRNA in (C) liver and (D) WAT of squirrels that received AdRR5 (black bars) or AdFGF21 (gray bars) for the indicated experiments corresponding to Figures 5–8. For all panels, data bars that do not share the same letter above the bar are significantly different from each other using ANOVA followed by Tukey's HSD (p<0.01). Errors bars show standard error of the mean. Abbreviations – n, number of animals; AUG, August active; TOR, torpor; IBA, interbout arousal; MAR, March active; WAT, white adipose tissue. (TIF) [file pone.0053574.s001.tif]
