# Supplementary material for: Metabolic Hormone FGF21 Is Induced in Ground Squirrels during Hibernation but Its Overexpression Is Not Sufficient to Cause Torpor
Source: PLoS One. 2013 Jan 2;8(1):e53574. doi: 10.1371/journal.pone.0053574 (PMC3534659; doi:10.1371/journal.pone.0053574)
Supplement: Table S1 — Sex of squirrels used in each experiment. (DOCX) [file pone.0053574.s002.docx]

**SUPPORTING TABLE**

| **Table S1. Sex of squirrels used in each experiment** | | | |
| --- | --- | --- | --- |
| Experiment | Sample Type | Female | Male |
| FGF21 mRNA (Fig. 2A); βKlotho and FGFR1 mRNA (Fig. 3) | Various tissues | 4 | 2 |
| FGF21 mRNA in liver (Fig. 2B); βKlotho mRNA in liver and WAT (Fig. S1A-B) | AUG | 3 | 3 |
|  | TOR | 5 | 1 |
|  | IBA | 3 | 3 |
|  | MAR | 3 | 3 |
| FGF21 protein in serum (Fig. 2C) | AUG | 4 | 4 |
|  | TOR | 4 | 5 |
|  | IBA | 3 | 5 |
|  | MAR | 3 | 5 |
| April Fed Adenoviral Infusions (Fig. 5) | Control | 4 | 4 |
|  | FGF21 | 4 | 4 |
| April Fasted Adenoviral Infusions (Fig. 6) | Control | 3 | 5 |
|  | FGF21 | 5 | 3 |
| August Fasted Adenoviral Infusions (Fig. 7) | Control | 4 | 4 |
|  | FGF21 | 4 | 3 |
| October Fasted Adenoviral Infusions (Fig.8) | Control | 4 | 3 |
|  | FGF21 | 6 | 2 |
